# Effects of Protein, Calcium, and pH on Gene Transcription, Cell-Envelope Peptidase Activity of *Lactococcus lactis* Strains, and the Formation of Bitter Peptides

**DOI:** 10.3390/foods10071588

**Published:** 2021-07-08

**Authors:** Benjamin Forler, Gudrun Horstmann, Johannes Schäfer, Christina Michel, Agnes Weiss, Timo Stressler, Lutz Fischer, Jörg Hinrichs, Herbert Schmidt

**Affiliations:** 1 Department of Food Microbiology and Hygiene, Institute of Food Science and Biotechnology, University of Hohenheim, Garbenstrasse 28, 70599 Stuttgart, Germany; benjamin.forler@uni-hohenheim.de (B.F.); christina.michel@uni-hohenheim.de (C.M.); agnes.weiss@uni-hohenheim.de (A.W.); 2 Department of Biotechnology and Enzyme Science, Institute of Food Science and Biotechnology, University of Hohenheim, Garbenstrasse 25, 70599 Stuttgart, Germany; gudrun.horstmann@uni-hohenheim.de (G.H.); timo.stressler@abenzymes.com (T.S.); Lutz.Fischer@uni-hohenheim.de (L.F.); 3 Department of Soft Matter Science and Dairy Technology, Institute of Food Science and Biotechnology, University of Hohenheim, Garbenstrasse 21, 70599 Stuttgart, Germany; J.Schaefer-3018@t-online.de (J.S.); j.hinrichs@uni-hohenheim.de (J.H.)

**Keywords:** concentrated fermented milk products, micro fresh cheese products, bitter peptides, transcriptional regulation, lactococcal cell-envelope peptidase PrtP activity

## Abstract

Calcium- and protein-rich fermented milk products, such as concentrated yoghurts and fresh cheeses, may contain undesired bitter peptides, which are generated by the proteolytic cleavage of casein. Up to now, it is not clear whether this process is caused by endogenous milk enzymes, such as plasmin and cathepsin D, or whether proteolytic enzymes from applied starter cultures, such as the lactococcal cell-envelope peptidase PrtP, are involved. A sensory analysis of fresh cheese products made from milk concentrates fermented with *prtP*-negative and -positive *Lactococcus lactis* strains revealed bitterness in the products fermented with *prtP*-positive *L. lactis* strains. Two *prtP*-positive strains, LTH 7122 and LTH 7123, were selected to investigate the effect of increased calcium concentrations (additional 5 mM and 50 mM CaCl_2_) at neutral (pH 6.6) and acidic (pH 5.5) pH-values on the transcription of the *prtP* gene and its corresponding PrtP peptidase activity in milk citrate broth (MCB). For both strains, it was shown that *prtP* transcription was upregulated only under slightly elevated calcium conditions (5 mM CaCl_2_) after 5 h of growth. In concordance with these findings, PrtP peptidase activity also increased. When higher concentrations of calcium were used (50 mM), *prtP* expression of both strains decreased strongly by more than 50%. Moreover, PrtP peptidase activity of strain LTH 7123 decreased by 15%, but enzymatic activity of strain LTH 7122 increased slightly during growth under elevated calcium concentrations (50 mM CaCl_2_). Fermentations of reconstituted casein medium with 3.4% (*w*/*v*) and 8.5% (*w*/*v*) protein and different calcium concentrations using strain LTH 7122 revealed no clear relationship between *prtP* transcription and calcium or protein concentration. However, an increase in PrtP peptidase activity under elevated protein and calcium conditions was observed. The activity increase was accompanied by increased levels of bitter peptides derived from different casein fractions. These findings could be a possible explanation for the bitterness in fermented milk concentrates that was detected by a trained bitter panel.

## 1. Introduction

The production of fermented milk products, such as curd cheese, strained (Greek-style) yogurt, or different types of fresh cheese, generates acid whey. Due to its high content of lactic acid, insoluble whey proteins, minerals such as calcium, enzymes, microorganisms and their metabolites, acid whey is difficult to process [1,2,3,4,5]. To cope with the increasing interest in fermented milk products containing a high protein content (>8%), such as Skyr or strained yogurt, and to bypass issues regarding acid whey, fermentation of calcium-rich concentrated milk, yielding a precious neutral “ideal whey” or permeate instead, seems to be an interesting alternative. Unfortunately, formation of bitter off-flavors in the fermented products has been found [5,6]. Since approximately 70% of milk calcium (valid for a native milk pH of about 6.6 and 20 °C) [7] is bound to the casein micelles, it is concentrated along with it by using membranes like ultra- or microfiltration [8]. The elevated protein and calcium content of concentrated milk, when used for fermentation, may alter the proteolytic activities of either milk-endogenous peptidases or those produced by the bacterial starter cultures [6].

Effective growth of lactic acid bacteria (LAB) depends strongly on their proteolytic system, as they are auxotrophic for several amino acids [9,10,11]. For this reason, most LAB express a membrane-anchored endopeptidase PrtP (alternatively cell-envelope peptidase) with an extracellular proteolytic domain that is responsible for the initial breakdown of surrounding milk proteins into oligopeptides [12,13,14]. PrtP-derived peptides can be easily transported into the cell by the oligopeptide permease Opp, which transports oligopeptides in the size of 4–35 amino acid residues. At least two other oligopeptide transporters, the dipeptide transporter Dpp and the di-/tripeptide transporter DtpT, which mainly transport smaller peptides [11], are also involved. The intracellular peptides are then further degraded into amino acids by synergistic effects of certain endo- and exopeptidases [10,15].

The cell-wall bound PrtP of *L. lactis* strains is encoded by the *prtP* gene, which is located on large plasmids (up to 80 kbp, see Table S1) and expressed as an inactive pro-form of about 200 kDa. After translocation through the cytoplasmic membrane, PrtP is activated by its membrane-anchored maturation protein PrtM [13,15,16,17,18]. The external part of PrtP harbors at least three calcium binding sites that support the stability of the peptidase as well as the maintenance of correct folding if calcium ions are present [19,20,21]. A classification system for lactococcal PrtP peptidases was proposed by Exterkate et al. in 1993 [22], where PrtP peptidases of different *L. lactis* strains were classified into seven groups based on the specificity of the peptidases towards the *α*_S1_-casein fragment (1–23). An eighth group was introduced in 1998 by Broadbent et al. [23]. As the specificity of the PrtP peptidases in *L. lactis* is very diverse, cleavage of the milk caseins by PrtP can yield more than 100 different peptides, among them important aroma components as well as bitter-tasting peptides [12,13,24,25,26]. The contribution of the starter culture on the formation of bitter peptides during the fermentation of concentrated milk (>8% protein) to produce fresh cheese was investigated by Sebald et al. [6]. In total, seventeen key bitter peptides derived from all casein fractions were detected. Especially, the peptides MAPKHKEMPFPKYPVEPF (derived from *β*-casein) and ARHPHPHLSFM (derived from *κ*-casein) were found to be primary contributors for sensory bitterness in fresh cheese made from concentrated milk [6].

The aim of the current study was to investigate the effects of increased calcium and protein concentrations on the transcription of the lactococcal PrtP peptidase gene as well as on the peptidase activity. Therefore, a micro fresh cheese model (µCoF) was used for the selection of bitterness-inducing *L. lactis* strains. These strains were used to investigate the influence of elevated calcium concentrations and a decreased pH-value on *prtP* transcription and PrtP peptidase activity in a milk citrate broth (MCB), which is described to initiate proteolytic activity in *Lacticaseibacillus casei* [27]. Moreover, one *prtP*-positive *L. lactis* strain was selected for fermentation in reconstituted casein medium with different protein and calcium concentrations. The impact of *prtP* transcription and PrtP peptidase activity on the formation of the aforementioned bitter peptides was quantified.

## 2. Materials and Methods

### 2.1. Strains and Plasmids

Bacterial strains and plasmids used in this study are listed in Table 1. *L. lactis* ssp. *cremoris* strain LTH 7122 and *L. lactis* ssp. *lactis* strain LTH 7123 were isolated from batch 3369251 of the starter culture F-DVS CC-06 (Christian Hansen Holding GmbH, Nienburg/Weser, Germany). *L. lactis* ssp. *lactis* strain LTH 7112 (syn. SL 124) and *L. lactis* ssp. *cremoris* strain LTH 7117 (syn. SC 134) were obtained from Sacco Srl, Cadorago, Italy. *L. lactis* strains were routinely grown in M17 broth over night and on M17 agar (both Merck KGaA, Darmstadt, Germany) for one to two days at 30 °C. Overnight cultures were prepared by inoculating M17 broth with colonies of the respective strain and incubating them at 30 °C without shaking under aerobic conditions.

*Escherichia coli* strains were routinely grown in Luria-Bertani (LB) broth [28]. Overnight cultures were prepared by inoculating LB broth with a single colony and incubating them at 37 °C, 180 rpm, in a rotary incubator.

### 2.2. Fermentation of Concentrated Milks

#### 2.2.1. Experimental Setup for Fermentation

In order to investigate the impact of the selected *L. lactis* strains on the fermentation characteristics and sensory properties of fresh cheese (CoF) made from concentrated milks obtained by microfiltration, a so-called micro fresh cheese system (µFCS) was used. The µFCS, which was described previously by Schäfer et al. [29], is an experimental setup at laboratory scale for fermentation of (concentrated) milk combined with pH monitoring and logging. For fermentation experiments, micellar casein powder (MCC60), which was prepared according to Schäfer et al. [29], was used. The contents of protein, dry matter, lactose, and calcium were 57.06 ± 0.12 g/100 g, 96.05 ± 0.12 g/100 g, 29.96 ± 0.85 g/100 g, and 19.82 ± 0.33 g/kg, respectively. The MCC60 powder was reconstituted (protein concentration: 8.5% (*w*/*w*); calcium content: 2.98 g/kg (74 mM; see Table 2)) with deionized water for 5 min at 50 °C and approx. 8.000 rpm using a rotor-stator mixer (Ultra-Turrax T50, IKA-Werke GmbH & Co. KG, Staufen, Germany). A batch-pasteurization (72 °C for 15 s) of the concentrate was conducted by means of a water bath adjusted to 85 °C. Pasteurization was stopped by cooling in ice water, and the concentrates were stored at 6 °C until use. The concentrated milk was warmed up to the fermentation temperature of 22.5 °C and transferred into sterilized plastic containers (volume = 400 mL) under a laminar flow cabinet. Each concentrate-filled container was inoculated with approx. 4.5 × 10^9^ colony-forming units (cfu) of each *L. lactis* strain. Containers were closed and sterilized pH probes (SE555X/2-NMSN; Knick Elektronische Messgeräte GmbH & Co. KG, Berlin, Germany) were inserted. 

The fermentation was conducted in a water bath (Kiss K 25; Peter Huber Kältemaschinenbau GmbH, Offenburg, Germany) at a temperature of 22.5 °C, and acidification was continuously monitored until a pH-value of 4.7 was achieved, or no change in pH-values for ≥4 h was observed. Obtained fermented concentrated milks and fresh cheese samples, respectively, were cooled to ≤6 °C and stored until use. The data were converted (Advantech-ADAM-6217, Sphinx Computer Vertriebs-GmbH, Laudenbach, Germany; MemoRail A1401, Knick Elektronische Messgeräte GmbH & Co. KG, Berlin, Germany), and pH-values as a function of fermentation time were recorded by means of a software (DAQ Factory Express, AzeoTech Inc., Ashland, MA, USA). The initial and final pH-values as well as the fermentation time were evaluated.

Furthermore, a CoF fresh cheese control sample was manufactured without fermentation of the pasteurized concentrated milk, i.e., direct acidification, as described previously by Schäfer et al. [30]. An amount of about 400 mL concentrate contained in a double-walled beaker was cooled to ≤2 °C in a water bath. During continuous stirring, the pH-value was adjusted to pH 4.7 by dropwise addition of 1 M citric acid (Carl Roth GmbH & Co. KG, Karlsruhe, Germany). To ensure a steady final pH, 10 min equilibration time were met and, if necessary, citric acid was added dropwise again. The acidified concentrated milk was filled into a sterilized plastic container (volume = 400 mL), and gel formation was initiated by means of warming using a water bath (15 °C) for a holding time of 2 h. The control CoF fresh cheese sample was cooled to ≤6 °C and stored until use. The manufacture of all CoF fresh cheeses was performed in duplicate.

As a reference fresh cheese sample, a commercially available fermented-concentrated (FCo) fresh cheese (Cremiger Quark mit frischem Joghurt, Berchtesgadener Land Chiemgau eG (D-BY 110 EG), Piding, Germany) was used containing 8.8 g/100 g protein and 1.26 g/kg calcium (31 mM) (Table 2).

#### 2.2.2. Sensory Evaluation of the Bitterness Level

The level of bitterness of the fresh cheese samples was evaluated after one week of storage by a trained sensory panel. The panel consisted of 25 members (male/female/diverse; smoker/non-smoker; age: approx. 22 to 58 years), and they were trained to evaluate the level of bitterness using caffeine solutions of 0.05, 0.1, 0.15, 0.175, 0.2, 0.25, and 0.3 g/L, respectively, according to the standard DIN ISO 3972:2013-12 [31] of the German Institute for Standardization (DIN)/International Organization for Standardization (ISO). All panelists could identify a caffeine concentration of at least 0.25 g/L as bitter, which fits the requirements recommended for a bitterness evaluation of food samples [31]. The training and evaluations were carried out at 22–25 °C in the Dr. Rainer-Wild sensory laboratory of the Institute of Food Science and Biotechnology, University of Hohenheim, Stuttgart, Germany, in separated booths.

In order to compare the fresh cheeses, they should have nearly the same appearance and consistency. Thus, prior to the sensorial evaluation, all fresh cheeses were mechanically treated with a hand blender (ESGE-Zauberstab M180, ESGE AG, Mettlen, Switzerland) at level two (*n* = 17,000 rpm) for 10 to 180 s until visibly smooth and homogenous masses, i.e., liquid to paste-like microgel suspensions, were obtained. The challenge was that some samples were liquid-like and contained gel particles; thus, a very short blending time of approximately 10 s had to be applied, and other samples appeared to be too thin. In order to evaluate the bitterness level of the fresh cheese samples, a polarized sensory positioning was carried out [32,33].

Depending on each trial, the number of panelists varied between 12 and 13. At each session, prior to the sensorial evaluation, two samples were served as reference for the panelists: (1) a commercially available FCo fresh cheese (Cremiger Quark mit frischem Joghurt, Berchtesgadener Land Chiemgau eG (D-BY 110 EG), Piding, Germany) and (2) a CoF fresh cheese which was manufactured from the reconstituted MCC60 powder and was fermented with the strain LTH 7122 at 22.5 °C. As this fresh cheese was ranked as very bitter by the sensorial panel, this fresh cheese was used as a bitter reference. 

The reference samples gave values of 0 (commercial FCo fresh cheese) and 4 (CoF fresh cheese fermented with LTH 7122) on a structured linear scale of 5 cm total length ranging from 0 to 5 (0: not bitter, 1: very slightly bitter, 2: slightly bitter, 3: bitter, 4: very bitter, 5: extremely bitter). 

The FCo fresh cheese product was chosen as a reference based on results of an in-house sensorial evaluation indicating that the bitterness level was ≤2 (slightly bitter) during 5 weeks of storage at approx. 6 °C, as described by Schäfer et al. [5].

Samples (approx. 20–30 g; temperature = approx. 10 °C) of each fresh cheese were served in plastic containers labeled with randomized 3-digit numbers. To neutralize any carry-over taste during the sensorial evaluation, tap water and matzo bread (Pico Food GmbH, Tamm, Germany) were served. As proposed by Sebald et al. [6], each panelist wore a nose-clip (Vitalograph GmbH, Hamburg, Germany) to prevent cross-modal interactions with other odorants during sample evaluation using the scale as mentioned above and at individual speed.

### 2.3. Isolation of *L. lactis* Plasmid DNA

Plasmid DNA was isolated according to the protocol of Anderson and McKay [34] with the following modifications: after the addition of lysozyme (10 mg/mL, SERVA Electrophoresis GmbH, Heidelberg, Germany), 20 µL RNase A (100 mg/mL, Qiagen GmbH, Hilden, Germany) was added to the reaction mixes, which were incubated at 37 °C for 60 min. Phenol, which was saturated with TE-buffer and chloroform-isoamyl alcohol (both Carl Roth GmbH & Co. KG, Karlsruhe, Germany), was used for the protocol. Purified plasmid DNA was dissolved in 30 µL of TE-buffer and analyzed by electrophoresis on 0.8% (*w*/*v*) agarose gels.

### 2.4. PCR Analysis of *prtP*

The presence of *prtP* was analyzed by amplifying a conserved region of 163 bp by PCR with the primers prtP_for (5′-CGC TTA GCC GCA TGT CTT TA-3′) and prtP_rev (5′-ACA GTC ACA TTG GCG AAA GT-3′). The PCR reaction mix consisted of 2.5 µL ThermoPol^®^ Buffer, 0.5 µL deoxynucleoside triphosphate (dNTP) mix (10 mM each) and 0.125 µL Taq DNA Polymerase (5,000 U/mL) (all New England Biolabs Inc., Ipswich, MA, USA), 0.5 µL of each primer (10 pmol/µL, Eurofins Genomics GmbH, Ebersberg, Germany), and 50 ng of plasmid DNA as template. Plasmid DNA of strains LTH 1657 and LTH 1418 was used as *prtP*-positive and *prtP*-negative controls, respectively. A no-template control (NTC) was included using sterile ultrapure water (GIBCO^TM^, Thermo Fisher Scientific Inc., Waltham, MA, USA) instead of template DNA. The volume was adjusted to 25 µL with sterile ultrapure water (GIBCO^TM^, Thermo Fisher Scientific Inc., Waltham, MA, USA). The PCR reaction was conducted in a T1 thermocycler (Biometra GmbH, Göttingen, Germany) with the following PCR conditions: initial denaturation, 95 °C, 5 min; 30 cycles of denaturation, 95 °C, 0.5 min, annealing, 51 °C, 0.5 min, and elongation, 68 °C, 0.5 min; and a final elongation at 68 °C, 5 min. The primer pair was constructed with the software Primer3web (version 4.1.0; http://primer3.ut.ee, accessed on 1 February 2018) using a conserved region of the *prtP* gene. This conserved region was identified by aligning known *prtP* sequences of different strains of *L. lactis* (Table S1) available at the National Center for Biology Information (NCBI).

### 2.5. Construction of a Plasmid Standard for the Absolute Quantification of *prtP* Gene Expression in a Quantitative Real-Time RT PCR (qPCR) Transcriptional Assay

For preparation of a plasmid standard for the absolute quantification of *prtP* transcription, the pGEM^®^-T Easy Vector System (Promega Corporation, Madison, WI, USA) was used. Therefore, the *prtP* PCR product of strain LTH 1657 (see Section 2.4) was purified with a QIAquick^®^ PCR Purification Kit (Qiagen GmbH, Hilden, Germany) following the corresponding purification protocol. The purified PCR products were cloned into plasmid pGEM^®^-T Easy following the pGEM^®^-T and pGEM^®^-T Easy Vector Systems technical manual (revised 12/18, TM042). Fifty nanogram of plasmid DNA and a three-fold molar excess of the PCR product was chosen for a ligation reaction, which was incubated at 4 °C overnight. Preparation of chemically competent *E. coli* DH5*α* cells and transformation procedures were carried out according to standard procedures [28]. Selection of transformants was carried out by blue-white screening on LB agar containing 100 µg/mL ampicillin, 0.5 mM isopropyl *β*-D-thiogalactopyranoside (IPTG), and 80 µg/mL 5-bromo-4-chloro-3-indoxyl-*β*-D-galactoside (X-Gal) (Carl Roth GmbH & Co. KG, Karlsruhe, Germany), according to the pGEM^®^-T and pGEM^®^-T Easy Vector Systems technical manual. Positive transformants were chosen for the isolation of plasmid DNA with a QIAprep Spin Miniprep Kit (Qiagen GmbH, Hilden, Germany), according to the handbook (July 2006). The resulting plasmid was analyzed by gel electrophoresis and designated pCM01.

### 2.6. Analysis of Lactococcal *prtP* Gene Expression during Casein Metabolism

#### 2.6.1. Preparation of Milk Citrate Broth, Calcium Solutions, and Reconstituted Casein Medium for Cultivation Experiments

For the preparation of 1 L MCB, 4.4 g skim milk powder (Oxoid Ltd., Basingstoke, UK) was mixed with deionized water and adjusted to 500 mL and pH 7.2. Additionally, 5 g glucose (Merck KGaA, Darmstadt, Germany), 8 g trisodium citrate dihydrate (Carl Roth GmbH & Co. KG, Karlsruhe, Germany), and 1 g yeast extract (Bacto^TM^, Fisher Scientific GmbH, Schwerte, Germany) were dissolved in deionized water and adjusted to 500 mL and pH 7.2. Both components were autoclaved separately, and the solutions were mixed under sterile conditions after cooling to 50 °C and stored at 4 °C. The final pH of the autoclaved medium was 6.6. This medium is designated in the following as basic MCB (pH 6.6).

In order to adjust the calcium content of the broth into the range of the CoF fresh cheese evaluated as bitter, sterile 0.5 M and 5 M calcium chloride stock solutions were used.

For cultivation in reconstituted casein medium with a modified calcium and protein content, the casein powders control MCC85 (protein content: 82.8 ± 1.89% (*w*/*w*); calcium content: 27.17 ± 0.01 g/kg) and calcium-reduced MCC85 (protein content: 85.23 ± 1.03% (*w*/*w*); calcium content: 15.25 ± 0.21 g/kg) [29] were used. In this work, the powder control MCC85 was designated as SdCa-mCN (slightly calcium-depleted micellar casein), and powder calcium-reduced MCC85 was designated as HdCa-mCN (highly calcium-depleted micellar casein). The powder HdCa-mCN is calcium-reduced by approx. 45.1% compared to casein powder SdCa-mCN. 

To get close to protein concentrations present in milk, the powders were reconstituted to a protein concentration of 3.4% (*w*/*v*), as described below. The respective calcium concentrations were 28 mM for SdCa-mCN (3.4% protein (*w*/*v*)) and 15 mM for HdCa-mCN (3.4% protein (*w*/*v*)). A high protein fermentation was conducted at a protein concentration of 8.5% (*w*/*v*), resulting in calcium concentrations of 70 mM for SdCa-mCN (8.5% protein (*w*/*v*)) and 38 mM for HdCa-mCN (8.5% protein (*w*/*v*)). Prior to fermentation, the solid casein powders were reconstituted in distilled and autoclaved water at 4 °C for 3–4 h, followed by a sterilization treatment at 95 °C for 30 min in a water bath. The sufficiency of the sterilization process was examined by use of plate count agar (0.5% (*w*/*v*) peptone, 0.25% (*w*/*v*) yeast extract, 0.1% (*w*/*v*) glucose, 1.6% (*w*/*v*) agar, pH 7). After sterilization, the reconstituted casein medium was stirred slowly over night at 4 °C and then used for the fermentation experiments on the next day. 

#### 2.6.2. Cultivation of *L. lactis* Strains in Milk Citrate Broth and Reconstituted Casein Medium with Different Calcium and Protein Concentrations 

Precultures in M17 broth were grown overnight (see Section 2.1) and the cells were harvested by centrifugation at 4255× *g*, resuspended in 0.1 M sodium phosphate buffer pH 7.2, and the optical density at 600 nm (OD_600_) was determined with a DU^®^ 730 UV/Vis Scanning Spectrophotometer (Beckman Coulter Inc., Brea, CA, USA). With this suspension, basic MCB or reconstituted casein medium (SdCa-/HdCa-mCN, each with 3.4% (*w*/*v*) and 8.5% (*w*/*v*) protein concentration) were inoculated to an OD_600_ of 0.1. In the experimental approaches with basic MCB and high calcium concentrations (in the range of the CoF fresh cheeses), 10 µL of the 0.5 M calcium chloride solution/mL was added to basic MCB (for additional 5 mM calcium chloride) and 10 µL of the 5 M calcium chloride solution/mL (for additional 50 mM calcium chloride) before addition of the cells.

After addition of 50 mM calcium chloride to basic MCB, the pH-value dropped from 6.6 to 5.5. In the approaches with MCB (pH 5.5), 3.4 µL 4 N HCl/mL was added before addition of the cells to adjust the pH-value to 5.5.

The cultures were then incubated under aerobic conditions at 30 °C without shaking for 24 h, and samples were taken hourly for serial dilution and were spread-plated in duplicate on M17 agar. The viable counts were determined after incubation at 30 °C for 24–48 h.

For RNA extraction, samples containing approx. 2 × 10^8^ cfu were taken in duplicate at the respective time points and treated with RNAprotect^®^ Bacteria Reagent (Qiagen GmbH, Hilden, Germany), according to the corresponding Handbook (January 2015) with the following modifications: centrifugation was performed at 4255× *g* and samples were stored at −70 °C until further processing.

#### 2.6.3. Sample Preparation for *prtP* Transcriptional Analysis

For RNA extraction, the samples (see Section 2.6.2) were thawed at room temperature and the RNA was isolated with the RNeasy^®^ Mini Kit (Qiagen GmbH, Hilden, Germany) following protocols 4 and 7 of the RNAprotect^®^ Bacteria Reagent Handbook with minor modifications. The samples were incubated at 37 °C with 750 rpm for 30 min on a thermomixer (protocol 4). The RNA was eluted (protocol 7) with 50 µL RNase-free water preheated to 37 °C and kept on ice for further processing or stored at −70 °C. Residual DNA within the RNA preparations was degraded with the Turbo DNA-free^TM^ Kit (Thermo Fisher Scientific Inc., Waltham, MA, USA) following the routine treatment protocol described in the TURBO DNA-free^TM^ Kit User Guide (TURBO^TM^ DNase Treatment and Removal Reagents, Revision G). The RNA integrity was examined by formaldehyde-based denaturing agarose gel electrophoresis. A total of 300 ng of RNA were reverse transcribed into cDNA with the iScript^TM^ Select cDNA Synthesis Kit (Bio-Rad Laboratories Inc., Hercules, CA, USA) following the instructions (10001023 Rev B) for the use of random primers. An additional approach with a no-reverse transcriptase control (-RT) was performed for each sample to detect residual DNA.

#### 2.6.4. Generation of Standard Curves for the Absolute Quantification of *prtP* Expression in the qPCR Transcriptional Assay

Plasmid pCM01 was serially diluted with sterile ultrapure water (GIBCO^TM^, Thermo Fisher Scientific Inc., Waltham, MA, USA) to obtain 6.0 × 10^2^ to 6.0 × 10^7^ copies/µL according to the technical manual “Creating Standard Curves with Genomic DNA or Plasmid DNA Templates for Use in Quantitative PCR” (Applied Biosystems, Waltham, MA, USA).

One microliter of each of pCM01 dilutions as well as 50 ng plasmid template DNA of the *L. lactis* type strain LTH 1657 were used in the respective *prtP*-qPCR assay (see Section 2.6.5) in order to confirm that the gene copy numbers were covered by the dynamic range of the plasmid standard curves. In the qPCR assays, the standard curves were automatically created with the threshold cycle values (C_t_ values) of the plasmid dilution series and the corresponding decadic logarithms of the starting quantity (either concentration or gene copy number) by the qPCR software (see Section 2.6.5). Three biological replicates with three technical replicates each were performed for proving the reproducibility of the standard plasmid curve, which all met the MIQE criteria [35].

#### 2.6.5. Establishment of a Real-Time PCR Assay for Absolute Quantification of *prtP* Transcription

For the qPCR assays, an iQ^TM^ 5 Multicolor Real-Time PCR Detection System with the corresponding software (Bio-Rad Laboratories Inc., Hercules, CA, USA) was used. PCR reactions with the *prtP* primer pair (see Section 2.4) were analyzed in gradient-PCR followed by melting curve analysis (Table 3) in order to prove the specificity of the PCR reaction and to determine the respective annealing temperatures. For these analyses, the SsoAdvanced Universal SYBR Green Supermix (Bio-Rad Laboratories Inc., Hercules, CA, USA) was used together with the primers (0.8 µL) and template DNA of the strain LTH 1657 (50 ng plasmid DNA) in a 20 µL reaction. The qPCR was set to the following conditions: initial denaturation, 95 °C, 5 min, and 40 cycles of denaturation (95 °C, 0.5 min), annealing (49.8 °C, 0.5 min), and elongation (68 °C, 0.5 min; collection of optical data for gene expression analysis).

### 2.7. Determination of PrtP Peptidase Activity

The determination of PrtP activity was conducted as described by Hebert et al. [36]. Samples (5 mL) were taken and harvested by centrifugation (4255× *g*, 4 °C, 10 min). Cells were washed with 0.9% (*w*/*v*) sodium chloride solution containing 10 mM of calcium chloride to avoid detachment of PrtP from the cell surface [37]. Subsequently, the washed cells were suspended in 50 mM sodium phosphate buffer (pH 7.0) and applied in the peptidase activity assay. The PrtP activity was determined by use of the synthetic substrate Suc-Ala-Ala-Pro-Phe-*p*NA (Bachem Holding AG, Bubendorf, Switzerland), which was dissolved in dimethylsulfoxide (12 mg/mL). The assay was conducted at a temperature of 30 °C in a thermoshaker. A volume of 30 µL of cell suspension was given to 200 µL of sodium phosphate buffer (pH 7), 100 µL of 5 M sodium chloride, and 20 µL of substrate solution. The substrate hydrolysis was stopped by addition of 100 µL of 80% (*v*/*v*) acetic acid. The assay’s incubation time was chosen with respect to the linear range of product formation as well as the assay’s limit of detection (data not shown). Precipitated protein was removed via centrifugation (17,950× *g*, 4 °C, 10 min), and 200 µL of the supernatant was transferred into a 96-well plate for measuring the absorption at 405 nm. One katal of PrtP peptidase activity was defined as the amount of PrtP that released 1 mol of *p*NA per second. Additionally, PrtP activity was standardized to its corresponding cfu (see Section 2.6.2).

### 2.8. Quantification of Key Bitter Peptides 

Certain key bitter peptides [6] were quantified within the fermentations of reconstituted casein medium. The cultivations in SdCa-mCN and HdCa-mCN medium (3.4% and 8.5% (*w*/*v*) protein content) were done in triplicates, and samples were taken after 5 and 24 h from every cultivation. The peptides were extracted as described by Sebald et al. [6]. Therefore, 1 ± 0.01 g of sample was extracted twice with 5 mL of 0.1% (*v*/*v*) formic acid by use of 3 ± 0.1 g of glass beads (∅ 3 mm). The internal standard angiotensin I (final concentration: 1 µg/mL) was given to the sample. After 3 × 20 s of mixing on a vortex, the sample was centrifuged (12,857× *g*, 4 °C, 10 min). The supernatant was adjusted to 10 mL in a measuring cylinder, sterile-filtered, and stored at −20 °C until further use. The concentration of bitter peptides was analyzed according to Sebald et al. [6] via mass-spectrometry by the Core Facility of the University of Hohenheim. The same peptides (peptides&elephants, Hennigsdorf, Germany) as published by Sebald et al. [6] were used for the calibration and quantification of the peptides within the fresh cheese samples. The determined peptide concentrations were used to calculate the dose-over-threshold-factor (DoT-factor) for every peptide. For that, the bitter taste thresholds published by Sebald et al. [6] of the peptides were used. On the basis of the DoT-factor, the influence of a peptide on the bitter taste reception can be estimated. At a DoT-factor of >1.0, a peptide can be assumed as being involved in the reception of bitterness in a product. An additive contribution can be performed by subthreshold peptides with DoT-factors of 0.1 ≤ DoT ≤ 1.0 [6]. 

### 2.9. Statistical Analysis

Of each data set, the means of three biological replicates were used for statistical analysis (of the data set HdCa-mCN 3.4% (*w*/*v*) for *prtP* transcriptional analysis, only two biological replicates could be used for analysis). Data sets of the different cultivation experiments were analyzed on normal distribution with the Anderson–Darling test. MCB data sets were compared to the reference (basic MCB) using an unpaired t-test. Fermentations in reconstituted casein medium were compared using analysis of variance (ANOVA). The variance homogeneity was identified using Brown–Forsythe test. If requirements were given, one-way ANOVA was performed using a post hoc Fisher’s least significant difference (LSD) test (*α* = 0.05). Statistical analysis was performed with the software OriginPro (version 2020, OriginLab Corporation, Northampton, MA, USA). Significant differences concerning the level of bitterness of the fresh cheeses were identified by conducting an ANOVA. If significant, a subsequent Fisher’s LSD test (*α* = 0.05) was applied. The software XLSTAT 2015 (version 2015.4.01.20575, Addinsoft, New York, NY, USA) was used.

## 3. Results

### 3.1. Fermentation of Concentrated Milks for the Production of CoF Fresh Cheese Samples and Sensory Evaluation

In order to produce fresh cheese (CoF) samples with *prtP*-positive and *prtP*-negative *L. lactis* strains for sensory evaluation, reconstituted casein medium with initial pH-values in a range of 6.6 to 6.8 were fermented until pH-values of 4.7 to 5.5 were reached (Table 2). The use of the *prtP*-negative strains LTH 7117 and LTH 7112 resulted in prolonged fermentation times of approx. 50 h and 66 h, respectively, as compared to fermentation with the *prtP*-positive strains LTH 7122 and LTH 7123 of approximately 27 h and 42 h, respectively, to achieve the desired pH-values under the same fermentation/growth conditions. Furthermore, *prtP*-negative strains did not acidify the concentrated milks to pH-values ≤ 5.1. Consequently, no homogenous gel formation was observed by visual inspection (Figure S1). Whereas the CoF fresh cheese obtained with LTH 7123 with a pH of 4.7 showed a homogenous gel, the CoF fresh cheese with LTH 7122 with same final pH showed a liquid-like (microgel) suspension with gel-particles contained therein.

**Figure 1:**
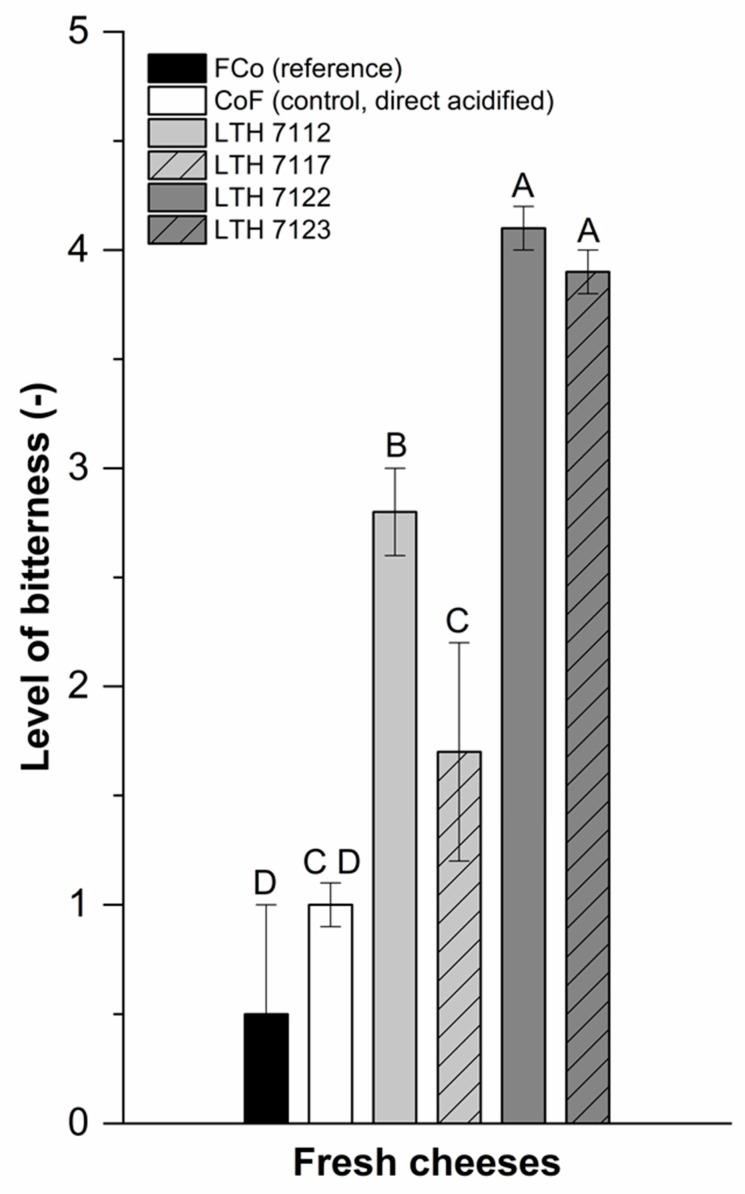
Level of bitterness of fermented-concentrated fresh cheese (FCo, black bar) used as a reference and concentrated-fermented (CoF) fresh cheeses manufactured by fermentation with *L. lactis* strains LTH 7112 (*prtP*-negative), LTH 7117 (*prtP*-negative), LTH 7122 (*prtP*-positive), LTH 7123 (*prtP-positive*), and without fermentation by direct acidification (CoF, control). 0: not bitter, 1: very slightly bitter, 2: slightly bitter, 3: bitter, 4: very bitter, 5: extremely bitter; A–D: bars with different uppercase letters are significantly different (*p* < 0.05). Values represent the means and standard deviations (i = 2, *n*_panelists_ ≥ 12).

Sensory evaluation indicated that CoF fresh cheese samples prepared with the *prtP*-negative strains LTH 7117 and LTH 7112 were perceived with low bitterness levels of 1.7 and 2.8, respectively, whereas the CoF fresh cheeses produced with *prtP*-positive strains LTH 7123 and LTH 7122 showed significantly higher bitterness levels of 3.9 and 4.1, respectively (Figure 1). CoF fresh cheeses obtained with the *prtP*-negative strains LTH 7117 and 7112 were perceived with higher bitterness levels than the commercial reference FCo fresh cheese with a bitterness level of 0.5.

In conclusion, sensory evaluation of fermented CoF fresh cheese samples using *prtP*-negative and *prtP*-positive strains revealed significantly higher bitterness levels than the non-fermented, directly acidified, CoF control sample (Figure 1).

The CoF-fresh-cheese sample fermented with strain LTH 7117 was also judged as slightly bitter. However, a significant difference in bitterness between the control CoF fresh cheese and the reference FCo fresh cheese was not noticed. Since the *L. lactis* strains LTH 7122 and LTH 7123 obviously were involved in the development of bitterness in the CoF fresh cheese, these strains were further investigated with molecular methods in terms of *prtP* transcription and PrtP activity.

### 3.2. Growth of *L. lactis* Strains LTH 7122 and LTH 7123 in Basic MCB and Quantification of *prtP* Transcription

**Figure 2:**
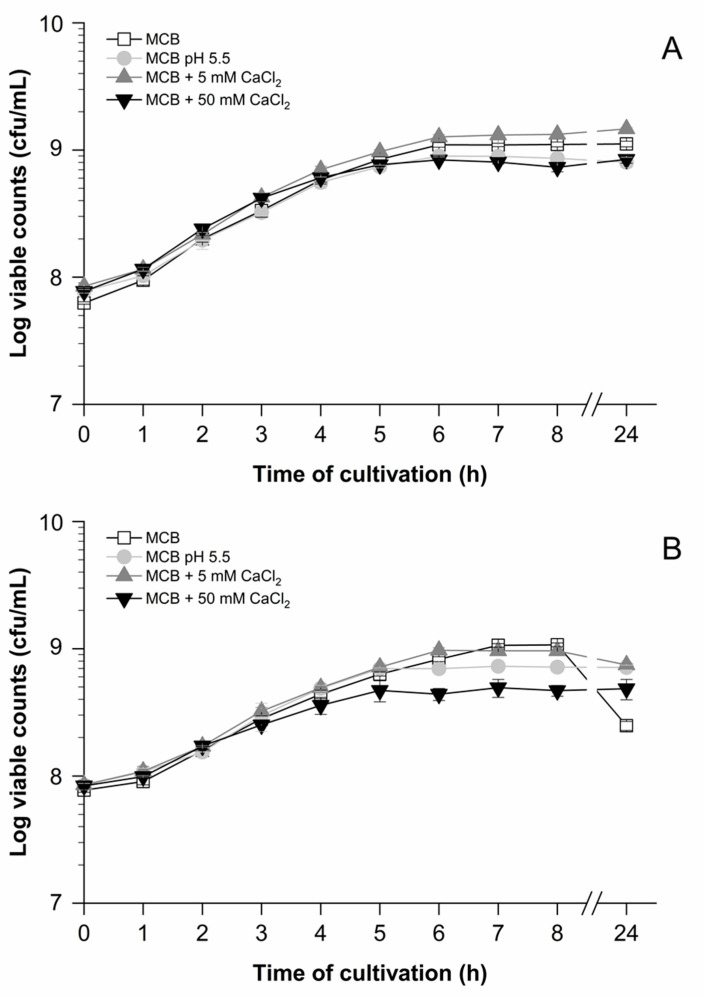
Semilogarithmic graph of growth curves of strains *L. lactis* LTH 7122 (**A**) and LTH 7123 (**B**) in different compositions of milk citrate broth (MCB). Cultivation was conducted for 24 h in basic MCB (pH 6.6), MCB (pH 5.5), basic MCB plus 5 mM calcium chloride, and basic MCB plus 50 mM calcium chloride at 30 °C without agitation. The viable counts (cfu/mL) were determined hourly. Data represent mean values and standard deviations from biological and technical duplicates.

**Figure 3:**
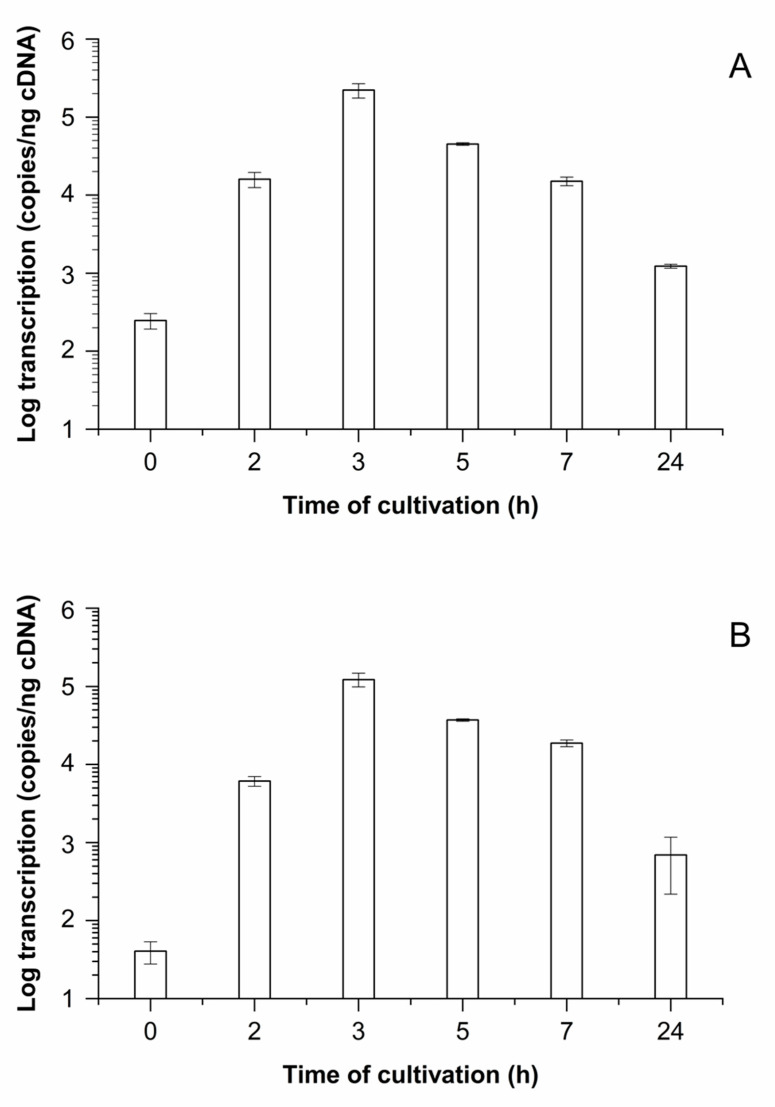
*prtP* transcription of strains *L. lactis* LTH 7122 (**A**) and LTH 7123 (**B**) during growth in basic MCB (pH 6.6). Cultivation was carried out for 24 h, and samples were collected at the respective time points. The mean values and standard deviations of three biological and two technical replicates each are shown.

In order to set up a system for quantitative analysis of *prtP* transcription under model conditions, basic MCB was used, which was previously described to initiate proteolytic activity in *Lacticaseibacillus casei* [27]. Initially, growth curves were generated with strains LTH 7122 and LTH 7123 in basic MCB (Figure 2, white squares) to evaluate the optimal conditions for qPCR. The cultures were started with a high inoculum of approx. 10^8^ cfu/mL to obtain sufficient RNA concentrations for qPCR analysis. Both strains showed similar growth characteristics in basic MCB and multiplied by approximately 1 log (Figure 2A,B, white squares). Only slight differences in viable counts were found. The *prtP* transcription levels (Figure 3A,B) were highest after 3 h of growth with 2.2 × 10^5^ copies/ng cDNA for LTH 7122 and 1.2 × 10^5^ copies/ng cDNA for LTH 7123, demonstrating slight differences between the strains. Afterwards, a continuous decrease in transcription levels (copies/ng cDNA) was observed with increasing cultivation time. Thus, *prtP* gene expression levels of both strains were highest in the early growth phase; therefore, 3 h and 5 h appeared as the most promising time points for subsequent analysis of the effects of different calcium concentrations and a low pH-value on *prtP* gene transcription.

### 3.3. Quantitative Analysis of *prtP* Transcription during Growth of Strains LTH 7122 and LTH 7123 in Basic MCB (pH 6.6), MCB (pH 5.5), and with Additional 5 mM or 50 mM Calcium Chloride

**Figure 4:**
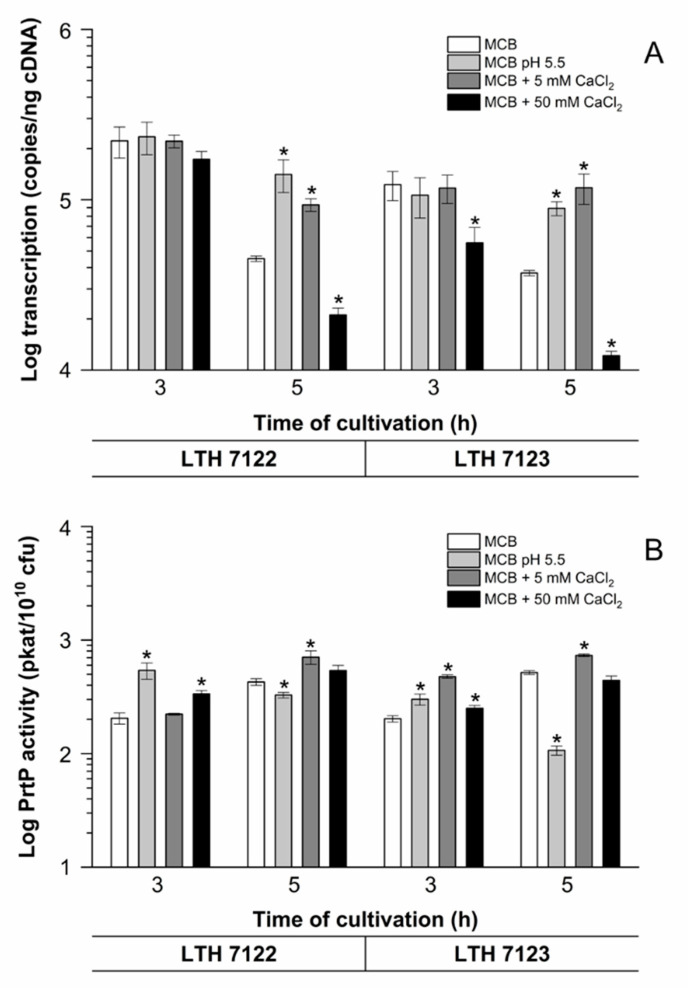
*prtP* transcription (**A**) and PrtP peptidase activity (**B**) of strains *L. lactis* LTH 7122 and LTH 7123 during growth in basic MCB (pH 6.6), MCB (pH 5.5), and basic MCB (pH 6.6) with addition of 5 mM or 50 mM calcium chloride. Samples were collected after 3 h and 5 h of cultivation. The mean values and standard deviations of three biological and two technical replicates each are shown. Asterisks (*) show statistical significance compared to the reference (basic MCB, pH 6.6) (*p* < 0.05).

As a prerequisite for quantitative expression analysis under certain growth conditions, growth curves of strains LTH 7122 (Figure 2A) and LTH 7123 (Figure 2B) were generated in basic MCB (pH 6.6), MCB (pH 5.5), and basic MCB with additional 5 mM or 50 mM calcium chloride. Since the supplementation of 50 mM calcium chloride to basic MCB reduced the initial pH from 6.6 to 5.5, control experiments were carried out with MCB (pH 5.5) in order to evaluate the effect of a lowered pH-value on growth and on *prtP* transcription levels (Figure 2 and Figure 4).

In the first 5 h after inoculation, both strains grew well with minor differences in generation time and viable counts (Figure 2). Whereas the decrease of the pH value to 5.5 had only a minor growth-delaying effect on strain LTH 7122 after 5 h of growth, the addition of 50 mM calcium chloride to basic MCB delayed the growth in terms of generation time and viable counts clearly in a strain-dependent manner (Figure 2A,B). However, the addition of 5 mM calcium chloride demonstrated a slightly growth-promoting effect, which was visible throughout the growth curve with strain LTH 7122 and after 5 h also with LTH 7123. These data supported the idea to measure *prtP* transcription at time points 3 h and 5 h after inoculation.

The *prtP* transcription levels of strains LTH 7122 and LTH 7123 during growth in basic MCB (pH 6.6), MCB (pH 5.5), and basic MCB (pH 6.6) with addition of 5 mM or 50 mM calcium chloride are summarized in Figure 4A.

The data obtained show a high transcription level of *prtP* after 3 h of growth in basic MCB (pH 6.6) for both strains and a lower transcription after 5 h of growth. For both strains, the *prtP* expression in MCB (pH 5.5) was similar to that in basic MCB (pH 6.6) after 3 h but was increased by 212% (LTH 7122) and 140% (LTH 7123) after 5 h of growth. A decrease of *prtP* expression levels during growth in basic MCB (pH 6.6) with additional 50 mM calcium chloride was observed with both strains (Figure 4A). After 3 h and 5 h of growth, the *prtP* transcription of strains LTH 7122 and LTH 7123 was reduced.

To investigate the effect of a lower calcium chloride concentration on *prtP* transcription, 5 mM calcium chloride was added to basic MCB (pH 6.6) (Figure 4A). In contrast to the results with 50 mM calcium chloride, *prtP* expression of strains LTH 7122 and LTH 7123 was affected in different ways. No effect was observed after 3 h, but transcription was increased by 107% in strain LTH 7122 and 217% in strain LTH 7123 after 5 h of growth. These data demonstrated that a high calcium concentration of 50 mM inhibits transcription, whereas the ten-times lower concentration promotes growth as well as *prtP* transcription in both tested strains.

### 3.4. Quantitative Analysis of PrtP Peptidase Activity during Growth of Strains LTH 7122 and LTH 7123 in Basic MCB (pH 6.6), MCB (pH 5.5), and with Additional 5 mM or 50 mM Calcium Chloride

The PrtP peptidase activity after 3 h of growth in basic MCB (pH 6.6) was approximately 200 pkat/10^10^ cfu and similar for both strains (Figure 4B). Accordingly, PrtP peptidase activity increased for both strains during the late exponential growth phase. The PrtP peptidase activity increased also during growth in MCB (pH 5.5) after 3 h of cultivation compared to the PrtP peptidase activity of cells grown in basic MCB (pH 6.6). However, after 5 h of growth, the PrtP peptidase activity of both strains was lower than at time point 3 h.

The addition of 5 mM CaCl_2_ to basic MCB (pH 6.6) led also to an increase in PrtP peptidase activity. For strain LTH 7122 a longer adaption to the conditions of elevated calcium was required, as the increased PrtP peptidase activity was determined just after 5 h of growth. In the beginning of the exponential growth at 3 h, this strain showed a PrtP peptidase activity similar to the PrtP peptidase activity of cells grown in basic MCB (pH 6.6) after 3 h of growth (Figure 4B). Strain LTH 7123 showed an increased PrtP peptidase activity from the beginning of exponential growth.

The PrtP peptidase activity increased for both strains during the exponential growth phase in basic MCB (pH 6.6) supplemented with 50 mM CaCl_2_. The PrtP peptidase activity of strain LTH 7123 was comparable to cells grown in basic MCB (pH 6.6), whereas PrtP peptidase activity of strain LTH 7122 increased to a low extent. At the end of exponential growth, PrtP peptidase activities were similar for both strains and comparable to the PrtP peptidase activity of cells grown in MCB medium.

### 3.5. Quantitative Analysis of *prtP* Transcription during Growth of Strain LTH 7122 in Reconstituted Casein Medium with Different Protein and Calcium Concentrations 

In order to confirm the results obtained in the experiments with MCB and to include the influence of elevated protein concentrations, a series of experiments was conducted using reconstituted casein medium with different protein and calcium concentrations (see Section 2.6.1). With this set of experiments, we wanted to shed light on the question of why the fermentation of milk concentrates, in contrast to the fermentation of milk, leads to strongly bitter-tasting fermentation products [5,6]. 

Strain LTH 7122 was selected for fermentation due to its strong bitterness promoting effect (see Section 3.1). Slightly calcium-reduced (SdCa-mCN) and highly calcium-reduced (HdCa-mCN) casein powder were used, and SdCa-mCN (protein: 3.4% (*w*/*v*); calcium: 28 mM) and HdCa-mCN casein medium (protein: 3.4% (*w*/*v*); calcium: 15 mM) were reconstituted to the native protein concentration of milk (3.4% (*w*/*v*) protein). Additionally, reconstituted SdCa-mCN (protein: 8.5% (*w*/*v*); calcium: 70 mM) and HdCa-mCN casein concentrates (protein: 8.5% (*w*/*v*); calcium: 38 mM) with an elevated protein concentration of 8.5% (*w*/*v*) were used.

**Figure 5:**
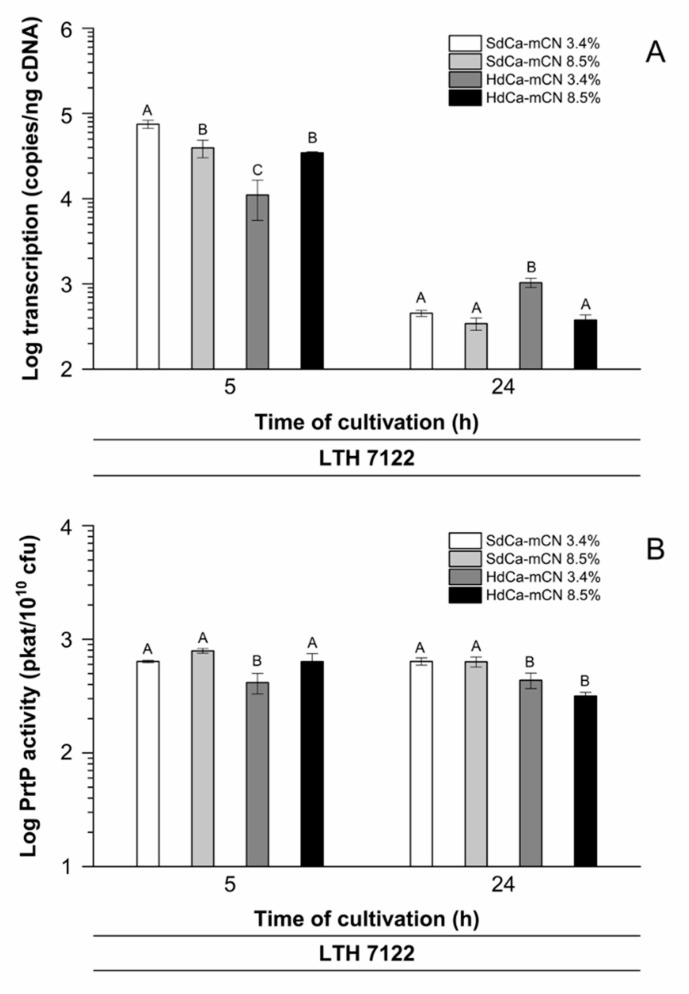
*prtP* transcription (**A**) and PrtP peptidase activity (**B**) of strain *L. lactis* LTH 7122 during cultivation in slightly calcium-reduced (SdCa-mCN) and highly calcium-reduced reconstituted casein medium (HdCa-mCN), each with a protein concentration of 3.4% (*w*/*v*) and 8.5% (*w*/*v*). Samples were collected after 5 h and 24 h of cultivation. The mean values and standard deviations of at least two biological replicates each are shown. A–C: bars with different uppercase letters are significantly different (*p* < 0.05).

The results of the transcriptional analyses of the *prtP* gene in reconstituted casein medium, differing in calcium and protein concentration, are shown in Figure 5A. When comparing the approaches of the protein-rich reconstituted casein media (protein: 8.5% (*w*/*v*)) after 5 h of cultivation, it was found that calcium reduction had little effect on the transcription of *prtP*. In contrast, when comparing the approaches with a native protein concentration of 3.4% (*w*/*v*), it was found that transcription was decreased by approximately 85% when using HdCa-mCN medium. Interestingly, the comparison of SdCa-mCN medium with a native (3.4% (*w*/*v*)) and an elevated (8.5% (*w*/*v*)) protein concentration revealed that transcription was decreased significantly by approximately 47% under an elevated protein concentration. The comparison of HdCa-mCN medium with a native (3.4% (*w*/*v*)) and an elevated (8.5% (*w*/*v*)) protein concentration showed the opposite effect: an increase of transcription by approximately 210% under an elevated protein concentration. 

After 24 h of cultivation, transcription was found to be decreased by more than 90% in all approaches compared with the respective 5 h approaches.

Thus, these results for strain LTH 7122 clearly showed that when reconstituted protein-rich casein concentrates (protein: 8.5% (*w*/*v*)) were used as the starting material for fermentation, there were no increased transcription rates of the *prtP* gene and also that a calcium reduction had little effect on transcription. Interestingly, even after 24 h of cultivation, there were no increased but significantly reduced transcription levels in the approaches with protein-rich concentrates compared to the respective approaches with a native protein concentration of 3.4% (*w*/*v*).

### 3.6. Quantitative Analysis of PrtP Peptidase Activity during Growth of Strain LTH 7122 in Reconstituted Casein Medium with Different Protein and Calcium Concentrations

*L. lactis* strain LTH 7122 was grown in media of reconstituted casein powders at two protein and calcium concentrations, respectively. The activity of PrtP was analyzed during exponential growth (5 h) and at the end of fermentation (24 h). Strain LTH 7122 was grown in reconstituted casein medium at a native protein concentration of 3.4% (*w*/*v*), which was compared to fermentation at an elevated protein concentration at 8.5% (*w*/*v*). At both protein concentrations, the calcium concentration was varied (Figure 5). 

In SdCa-mCN medium (protein: 3.4% (*w*/*v*); calcium: 28 mM), a constant PrtP activity of 637 ± 15 and 638 ± 47 pkat/10^10^ cfu was determined. In SdCa-mCN medium under elevated protein and calcium conditions (protein: 8.5% (*w*/*v*); calcium: 70 mM), the PrtP activity was increased to 789 ± 38 pkat/10^10^ cfu during the exponential growth phase of LTH 7122. After 24 h, a similar PrtP activity as in SdCa-mCN (protein: 3.4% (*w*/*v*); calcium: 28 mM) was determined (633 ± 63 pkat/10^10^ cfu). In HdCa-mCN medium, the calcium concentration was reduced by 48.6% compared to native casein. To HdCa-mCN medium (protein: 3.4% (*w*/*v*); calcium: 15 mM), the PrtP activity reacted in the same manner as in SdCa-mCN medium and kept constant during exponential growth (5 h) as well as at the end of fermentation (24 h). However, the level of PrtP activity was reduced (416 ± 86 pkat/10^10^ cfu and 436 ± 68 pkat/10^10^ cfu, respectively). An increase in protein and calcium concentration of HdCa-mCN (protein: 8.5% (*w*/*v*); calcium: 38 mM) led to a PrtP activity comparable to SdCa-mCN (protein: 3.4% (*w*/*v*); calcium: 28 mM) during exponential growth (636 ± 8 pkat/10^10^ cfu), while at the end of fermentation, the PrtP activity declined (316 ± 25 pkat/10^10^ cfu), as seen for SdCa-mCN medium at elevated protein conditions (protein: 8.5% (*w*/*v*); calcium: 70 mM). 

### 3.7. Quantitative Analysis of Key Bitter Peptides Formed during Growth of Strain LTH 7122 in Reconstituted Casein Medium with Different Protein and Calcium Concentrations

As seen in the sensorial evaluation of CoF fresh cheese described before, strain LTH 7122 showed a capability to produce bitterness during fermentation. Therefore, samples from fermentation experiments described before were examined for their concentration of bitter peptides (Table 4). Based on the study of Sebald et al. [6], the seventeen published key bitter peptides derived from different casein fractions were used for that investigation. 

Sixteen of the seventeen key peptides were found in the fermentation samples although at low concentrations, in most cases. In SdCa-mCN medium (protein: 3.4% (*w*/*v*); calcium: 28 mM), the concentration of peptides increased in the progress of fermentation and as well as at elevated protein and calcium conditions (protein: 8.5% (*w*/*v*); calcium: 70 mM). Especially the peptides VLPVPQ, MAPKHKEMPFPKYPVEPF (both derived from *β*-casein), and ARHPHPHLSFM (derived from *κ*-casein) were determined in higher concentrations. In comparison to that, in HdCa-mCN (protein: 3.4% (*w*/*v*); calcium: 15 mM) the peptide concentrations were found in comparable or lower concentrations except for the peptides MAPKHKEMPFPKYPVEPF and KVLPVPQKAVPYPQ, which derived from *β*-casein. The increase in protein and calcium content in casein concentrate HdCa-mCN (protein: 8.5% (*w*/*v*); calcium: 38 mM) led to higher peptide concentrations than in HdCa-mCN medium (protein: 3.4% (*w*/*v*); calcium: 15 mM). Compared to SdCa-mCN medium (protein: 3.4% (*w*/*v*); calcium: 28 mM), most peptide concentrations were increased although the calcium concentration was at a comparable level. The highest peptide concentrations were determined for ARHPHPHLSFM in the fermentation samples. This peptide derived from *κ*-casein and is also stated to be a primary contribution to bitterness. 

Nevertheless, the DoT-factors were < 0.1 for the majority of the samples except for ARHPHPHLSFM, which additionally showed the highest DoT-factor of 0.7 in SdCa-mCN (protein: 8.5% (*w*/*v*); calcium: 70 mM).

## 4. Discussion

In the current study, *prtP* transcription and PrtP peptidase activity of two *prtP*-positive single starter strains that were isolated from a commercial starter culture were analyzed in MCB and reconstituted casein media with various calcium and protein concentrations. The two strains were chosen since the sensory analyses of fermented concentrated milk (containing approximately 74 mM calcium) revealed that both strains induce a strong sensory bitterness in the fermented dairy products.

Our findings that the CoF-fresh cheese sample fermented with the *prtP*-negative strain LTH 7112 was significantly more bitter than the CoF-control indicates that even *prtP*-negative strains may be able to induce bitterness in CoF fresh cheese to some extent. Even the control CoF fresh cheese was perceived as very slightly bitter (bitterness level of 1.0), which was not completely in line with results of Poulsen and Mogensen [38], who reported that an ultrafiltration retentate, which was acidified with glucono-*δ*-lactone, was perceived as not bitter. The slight differences between both control and reference samples show that it was difficult for the panelists to differentiate between those low bitter-tasting samples. 

The growth curve analyses in different compositions of MCB medium revealed that the addition of 5 mM calcium chloride to basic MCB caused a growth promoting and the addition of 50 mM calcium chloride a growth-impairing effect on both strains LTH 7122 and LTH 7123. Since growth rates of LAB in milk are strongly dependent on casein proteolysis [12,14,39,40], similar effects regarding the transcription levels and the enzymatic activity of the cell-envelope peptidase PrtP were expected. Since proteolysis by the PrtP peptidase activity leads to various casein-derived oligopeptides, among them also bitter tasting peptides [12,13,24,25,26], an upregulation of *prtP* could also be considered as a bitterness-increasing process.

The results of the transcriptional analyses in basic MCB coincide with the previously described findings that PrtP peptidase activity is crucial for growth in milk [39] and that the expression level also depends on the growth phase and growth rate [41,42,43]. 

In the experiments using different compositions of MCB, elevated concentrations of CaCl_2_ (50 mM) were shown to decrease *prtP* transcription, which is in contrast to the initial hypothesis that high levels of calcium lead to an upregulation of *prtP*. However, upon the addition of 5 mM calcium chloride, higher transcription levels of *prtP* were observed. 

In contrast to the transcriptional analyses, it could be shown that the addition of calcium to basic MCB resulted generally in a higher PrtP activity during bacterial growth. A similar behavior was already published by Exterkate [44], who stated that the PrtP peptidase activity of *L. lactis* ssp. *cremoris* strain AM_1_ depends on the calcium concentration of the surrounding medium. In their study, an addition of 5 mM calcium to the growth medium increased the PrtP peptidase activity compared to cells grown on the same medium without the added calcium. It was found that the addition of 50 mM also increased the proteolytic activity of the PrtP peptidase although to a lesser extent than 5 mM.

The cultivation experiments in reconstituted casein medium with 3.4% (*w*/*v*) and 8.5% (*w*/*v*) protein content revealed that *prtP* transcription was not clearly increased when strain LTH 7122 was grown in protein-rich casein concentrates. Additionally, high calcium concentrations also did not clearly increase *prtP* transcription. These results suggest that the previously observed increased bitterness in calcium-rich, fermented dairy products, which was also described by Schäfer et al. and Sebald et al. [5,6], cannot be explained by sheer alterations in *prtP* transcription due to high calcium or protein concentrations. 

In contrast to the transcription levels, PrtP peptidase activity was mainly increased at elevated calcium and protein concentrations. These results are in line with the study of Exterkate [44], who observed increased activity at elevated calcium conditions. The quantification of key bitter peptides within those samples revealed higher peptide concentrations at elevated protein- and calcium concentrations. Two of those peptides (MAPKHKEMPFPKYPVEPF and ARHPHPHLSFM) are known to exhibit a low taste threshold and thus can be assumed to have a strong impact on bitterness perception [6]. Especially for the *κ*-casein derived peptide ARHPHPHLSFM, concentrations up to 20 µmol/kg were determined after 24 h of fermentation in 8.5% (*w*/*v*) protein at a calcium concentration of 70 mM. For the other investigated bitter peptides, a similar increase in concentration was observed under increased protein concentrations and furthermore with increasing calcium concentrations. Although the concentrations of the peptides alone are below the taste thresholds [6], it is possible that the combination of different bitter peptides has a synergistic effect, and the taste perception is yet bitter for the consumer. The observed strain dependency of our results is in accordance with results of Meijer et al. [42], who reported a strain-dependent regulation of lactococcal PrtP peptidase activity.

## 5. Conclusions

The sensorial evaluation revealed a perceivable bitterness in high-protein fresh cheese fermented with *prtP*-positive *L. lactis* strains, whereas *prtP*-negative *L. lactis* strains did not introduce bitterness. Although the *prtP*-gene expression was not upregulated by highly elevated calcium or protein concentrations, the PrtP peptidase activity was increased, resulting in higher concentrations of bitter peptides within the investigated casein media. Thus, the problem of bitterness in high-protein dairy products might be caused by certain *L. lactis* strains and their extracellular endopeptidase PrtP. A sensible selection and combination of *prtP*-positive and -negative starter strains might be a useful tool to overcome the difficulty of bitterness in high-protein dairy products and should be investigated in future studies.

## Figures and Tables

**Table 1:** Strains and plasmids used in this study.

Strain or Plasmid	Subspecies and Geno-/Phenotype	Reference or Source
***L. lactis***		
LTH 1657	ssp. *cremoris* **^T^**, *prtP*-positive	ATCC 19257
LTH 1418	ssp. *lactis* **^T^**, *prtP*-negative	ATCC 19435
LTH 7122	ssp. *cremoris*, *prtP*-positive	Christian Hansen Holding GmbH, Nienburg/Weser, Germany, this study
LTH 7123	ssp. *lactis*, *prtP*-positive	Christian Hansen Holding GmbH, Nienburg/Weser, Germany, this study
LTH 7117	ssp. *cremoris*, *prtP*-negative	Sacco Srl, Cadorago, Italy
LTH 7112	ssp. *lactis*, *prtP*-negative	Sacco Srl, Cadorago, Italy
***E. coli***		
DH5*α*	*tonA lacZΔ*M15 *endA1 recA1 thi-1 supE44 phoA gyrA96 hsdR17 Δ*(*lacZYA*-*argF*)*U169 relA1*	Invitrogen, Carlsbad, CA, USA
DH5*α*/pCM01	Donor for plasmid pCM01	This study
**Plasmids**		
pGEM^®^-T Easy	Vector for cloning of PCR products	Promega Corporation, Madison, WI, USA
pCM01	163 bp *prtP* PCR product, cloned into pGEM^®^-T Easy	This study

**^T^** = type strain; ATCC = American Type Culture Collection (https://www.lgcstandards-atcc.org/en/Products/Cells_and_Microorganisms/Bacteria.aspx, accessed on 31 May 2021).

**Table 2:** Chemical composition and fermentation characteristics of (reference) fermented-concentrated and concentrated-fermented fresh cheeses manufactured by means of fermentation and without fermentation (control, direct acidified). Values represent the means and standard deviations (i = 2, *n* = 2).

Fresh Cheese Sample	Strain	*prtP*	Protein (%, *w*/*w*)	Calcium ^a^ (g/kg)	Inital pH (−)	Final pH (−)	Fermentation Time (h:min)	Texture ^c^
FCo (reference)	blend	positive and negative strains	8.8 ^b^	1.26 ^b^	n.i.	4.5 ± 0.1	n.i.	Paste-like
CoF (control, directly acidified)	n.d.	n.d.	8.5	2.98	6.8 ± 0.1	4.7 ± 0.1	n.d.	Gel (Paste-like after mechanical treatment)
CoF	LTH 7112	negative	8.5	2.98	6.6 ± 0.1	5.1 ± 0.1	65:33 ± 13:15	Liquid, gel particles present
CoF	LTH 7117	negative	8.5	2.98	6.7 ± 0.1	5.5 ± 0.1	50:11 ± 2:59	Liquid, gel particles present
CoF	LTH 7122	positive	8.5	2.98	6.7 ± 0.1	4.7 ± 0.3	41:47 ± 0:1	Liquid, gel particles present
CoF	LTH 7123	positive	8.5	2.98	6.7 ± 0.1	4.7 ± 0.1	27:11 ± 0:11	Gel (Paste-like after mechanical treatment)

^a^ data was calculated by means of values from chapter 2.2; ^b^ according to manufacturer’s specification; ^c^ the texture of the fermented concentrates was evaluated prior to the mechanical treatment only via the naked eye. n.i.: not indicated by the manufacturer. n.d.: no data available. CoF: concentrated-fermented. FCo: fermented-concentrated.

**Table 3:** Cycling programs of the gradient-PCR and melt curve analysis for the *prtP*-qPCR establishment.

Reaction Step	Temperature (°C)	Time (s)	Cycles
Initial denaturation	95	300	1
Denaturation	95	30	40
Annealing	59.0, 58.5, 57.3, 55.4, 52.9, 51.1, 49.8, 49.0	30	
Elongation *	68	30	
Denaturation	95	60	1
Renaturation	25	60	1
Denaturation **	25 → 95 (increase 0.5 per cycle)	10	141

* Collection of optical data for the optimization of the annealing temperature. ** Collection of optical data for the melt curve analysis.

**Table 4:** Concentrations of key bitter peptides formed during cultivation of strain LTH 7122 in reconstituted casein medium with different protein and calcium concentrations and corresponding DoT factors. -: peptide concentrations too low for detection.

Derived From	Peptide Sequence	Peptide Concentration (µmol/kg) (DoT-Factor (–))							
		3.4% (*w*/*v*) SdCa-mCN		8.5% (*w*/*v*) SdCa-mCN		3.4% (*w*/*v*) HdCa-mCN		8.5% (*w*/*v*) HdCa-mCN	
		5 h	24 h	5 h	24 h	5 h	24 h	5 h	24 h
*α*_s1_-Casein	VAPFPEVPGKE	0.15 ± 0.01 (<0.1)	0.58 ± 0.01 (<0.1)	0.25 ± 0.01 (<0.1)	1.36 ± 0.10 (<0.1)	0.13 ± 0.00 (<0.1)	0.50 ± 0.01 (<0.1)	0.13 ± 0.00 (<0.1)	1.78 ± 0.02 (<0.1)
	VFGKEKVNEL	-	0.13 ± 0.01 (<0.1)	0.03 ± 0.01 (<0.1)	0.35 ± 0.40 (<0.1)	-	0.16 ± 0.00 (<0.1)	-	1.44 ± 0.08 (<0.1)
	DIKQM	-	-	-	-	-	-	-	-
	EIVPNS(PHOS)VEQK	-	-	0.88 ± 0.08 (<0.1)	0.83 ± 0.20 (<0.1)	-	-	0.59 ± 0.02 (<0.1)	2.30 ± 0.30 (<0.1)
	IQKEDVPS	-	-	-	0.09 ± 0.01 (<0.1)	-	-	-	0.14 ± 0.01 (<0.1)
*β*-Casein	TQTPVVVPPFLQPE	-	-	0.05 ± 0.01 (<0.1)	0.58 ± 0.01 (<0.1)	-	0.03 ± 0.00 (<0.1)	-	0.91 ± 0.02 (<0.1)
	MAPKHKEMPFPKYPVEPF	0.02 ± 0.00 (<0.1)	0.36 ± 0.04 (<0.1)	0.05 ± 0.01 (<0.1)	0.87 ± 0.10 (<0.1)	-	0.61 ± 0.01 (<0.1)	0.06 ± 0.00 (<0.1)	1.33 ± 0.18 (<0.1)
	LHLPLP	-	0.51 ± 0.10 (<0.1)	-	1.01 ± 0.02 (<0.1)	-	0.15 ± 0.01 (<0.1)	-	-
	LHLPLPLL	-	-	-	-	-	-	-	-
	HLPLPLLQ	-	0.23 ± 0.01 (<0.1)	-	0.78 ± 0.02 (<0.1)	-	0.26 ± 0.01 (<0.1)	-	0.68 ± 0.01 (<0.1)
	KVLPVPQKAVPYPQ	0.06 ± 0.01 (<0.1)	0.10 ± 0.01 (<0.1)	0.09 ± 0.02 (<0.1)	0.30 ± 0.04 (<0.1)	-	0.28 ± 0.01 (<0.1)	0.16 ± 0.01 (<0.1)	1.29 ± 0.04 (<0.1)
	VLPVPQ	0.57 ± 0.10 (<0.1)	2.79 ± 0.40 (<0.1)	1.61 ± 0.20 (<0.1)	6.82 ± 1.10 (<0.1)	0.21 ± 0.01 (<0.1)	1.14 ± 0.04 (<0.1)	0.30 ± 0.02 (<0.1)	3.38 ± 0.12 (<0.1)
*κ*-Casein	FFSDKIAK	-	-	-	-	-	-	-	-
	YQQPVAL	-	4.30 ± 0.16 (<0.1)	-	0.08 ± 0.02 (<0.1)	-	0.11 ± 0.00 (<0.1)	-	0.31 ± 0.00 (<0.1)
	ARHPHPHLSFM	0.66 ± 0.01 (<0.1)	11.20 ± 1.52 (0.4)	0.99 ± 0.01 (<0.1)	19.93 ± 1.40 (0.7)	0.21 ± 0.01 (<0.1)	1.89 ± 0.13 (<0.1)	0.30 ± 0.04 (<0.1)	2.80 ± 0.33 (0.1)
	AIPPKKNQDKTEIPT- INTIASGEPT	-	-	-	0.22 ± 0.01 (<0.1)	-	-	-	-
	INTIASGEPT	-	-	-	-	-	-	-	-

## Data Availability

Data available upon request from corresponding author.

## Supplementary Materials

The following are available online at https://www.mdpi.com/article/10.3390/foods10071588/s1, Figure S1. Photos of fermented-concentrated fresh cheese (FCo) used as a reference and concentrated-fermented (CoF) fresh cheeses manufactured by means of fermentation with *L. lactis* strains LTH 7112 (*prtP*-negative), LTH 7117 (*prtP*-negative), LTH 7122 (*prtP*-positive), LTH 7123 (*prtP*-positive), and without fermentation by means of direct acidification (CoF, control) before a mechanical treatment of their gels. ^1^The FCo fresh cheese was already mechanically treated during manufacture; Table S1. Overview of the specific sequences and their location used for the *prtP* sequence alignment for determination of a conserved *prtP* region for primer design.
