# Supplementary material for: Effects of Protein, Calcium, and pH on Gene Transcription, Cell-Envelope Peptidase Activity of Lactococcus lactis Strains, and the Formation of Bitter Peptides
Source: Foods. 2021 Jul 8;10(7):1588. doi: 10.3390/foods10071588 (PMC8307170; doi:10.3390/foods10071588)
Supplement: Supplementary file 1 [file foods-10-01588-s001.zip › foods-1264809-supplementary.pdf]

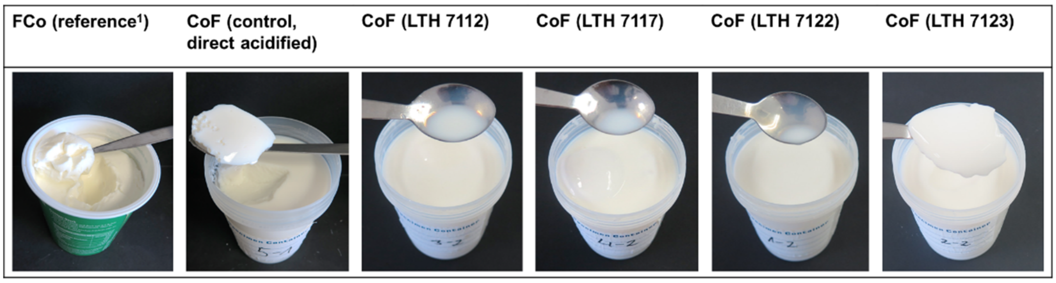

Figure S1. Photos of fermented-concentrated fresh cheese (FCo) used as a reference and concentrated-fermented (CoF) fresh cheeses manufactured by means of fermentation with *L. lactis* strains LTH 7112 (*prtP*-negative), LTH 7117 (*prtP*-negative), LTH 7122 (*prtP*-positive), LTH 7123 (*prtP*-positive), and without fermentation by means of direct acidification (CoF, control) before a mechanical treatment of their gels. [1]The FCo fresh cheese was already mechanically treated during manufacture.

Table S1. Overview of the specific sequences and their location used for the *prtP* sequence alignment for determination of a conserved *prtP* region for primer design.

Strain	NCBI accession number	***prtP*** **coding plasmid and gene region**
Lactococcus lactis subsp. *cremoris* NCDO712	FJ649478.1	pLP712 (55,395 kbp), 7137..13025
Lactococcus lactis subsp. *lactis* 229	CP016695.1	p229B (33,280 kbp), 19843..25731
Lactococcus lactis subsp. *cremoris* UC509.9	CP003160.1	pCIS6 (38,673 kbp), 25236..31160
Lactococcus lactis subsp. *cremoris* 158	CP016688.1	p158D (33,287 kbp), 21293..25768
Lactococcus lactis subsp. *cremoris* SK11	CP000428.1	plasmid 3 (74,750 kbp), 45329..51217
Lactococcus lactis subsp. *lactis* UC063	CP016715.1	pUC063A (75,962 kbp), 34100..39982
